# miRNA-223 upregulated by MYOD inhibits myoblast proliferation by repressing IGF2 and facilitates myoblast differentiation by inhibiting ZEB1

**DOI:** 10.1038/cddis.2017.479

**Published:** 2017-10-05

**Authors:** Guihuan Li, Wen Luo, Bahareldin A Abdalla, Hongjia Ouyang, Jiao Yu, Fan Hu, Qinghua Nie, Xiquan Zhang

**Affiliations:** 1 Department of Animal Genetics, Breeding and Reproduction, College of Animal Science, South China Agricultural University, Guangzhou 510642, Guangdong Province, China; 2 Guangdong Provincial Key Lab of Agro-Animal Genomics and Molecular Breeding, and Key Lab of Chicken Genetics, Breeding and Reproduction, Ministry of Agriculture, South China Agricultural University, Guangzhou 510642, Guangdong Province, China; 3 Department of Cell Biology, School of Basic Medical Sciences, Southern Medical University, Guangzhou 510515, China

## Abstract

Skeletal muscle differentiation can be regulated by various transcription factors and non-coding RNAs. In our previous work, miR-223 is differentially expressed in the skeletal muscle of chicken with different growth rates, but its role, expression and action mechanism in muscle development still remains unknown. Here, we found that MYOD transcription factor can upregulate miR-223 expression by binding to an E-box region of the *gga-miR-223* gene promoter during avian myoblast differentiation. *IGF2* and *ZEB1* are two target genes of miR-223. The target inhibition of miR-223 on *IGF2* and *ZEB1* are dynamic from proliferation to differentiation of myoblast. miR-223 inhibits *IGF2* expression only in the proliferating myoblast, whereas it inhibits *ZEB1* mainly in the differentiating myoblast. The inhibition of *IGF2* by miR-223 resulted in the repression of myoblast proliferation. During myoblast differentiation, miR-223 would be upregulated owing to the promoting effect of MYOD, and the upregulation of miR-223 would inhibit *ZEB1* to promote myoblast differentiation. These results not only demonstrated that the well-known muscle determination factor MYOD can promote myoblast differentiation by upregulate miR-223 transcription, but also identified that miR-223 can influence myoblast proliferation and differentiation by a dynamic manner regulates the expression of its target genes.

MicroRNAs (miRNAs) are small non-coding RNA of 20–25 nucleotides that mainly transcript from introns or intergenic regions, having critical roles in the regulation of gene expression at posttranscriptional levels.^1^ It have been reported that miRNAs widely participate in chicken embryo growth, maturation, muscle cell proliferation and differentiation, cell migration, dosage compensation effect of Z chromosome and a series of life activities.^2, 3, 4, 5, 6, 7^ In our preliminary study, we have used high-throughput RNA sequencing to study breast muscle transcriptome in Recessive White Rock (fast-growing chicken) and Xinghua chicken (slow-growing chicken), and pointed out that miR-223 can be used as a candidate gene associating with broiler growth.^8^

The miR-223 is highly conserved among vertebrates. As the first miRNA identified in human hemopoietic system,^9^ miR-223 was confirmed to participate in the regulation of hematopoietic cell proliferation and differentiation,^10^ and become a research hotspot. There are some competitive binding sites for NFI-A and C/EBP alpha in the upstream of *miR-223*. C/EBP alpha enhances *miR-223* transcription activity, whereas NFI-A inhibits the transcription activity of *miR-223*.^10^ The upregulation of miR-223 expression will promote granulocyte and megakaryocyte generation, and the decline of miR-223 expression will cause hematopoietic stem cells to differentiate into erythrocyte.^11, 12^ In addition, miR-223 was confirmed to regulate cell proliferation, differentiation, migration and signal transduction in mammals.^13^ However, the roles of miR-223 in muscle development still remain unclear.

Insulin-like growth factor-2 (IGF2) is a member of insulin-like growth factor family that can play a role in all insulin-activated target cells.^14^ It is well-known that IGF2 can be acted as the intermediate messenger of growth hormone, which can transfer the growth hormone to target organs, and regulate the development of organisms.^15^ IGF2 is conserved during evolution, the gene structure and exons location in chicken are similar to that in mammal, although the non-coding sequences were very different.^16^ In mammals, IGF2 is a kind of multifunctional cell regulation factor that has an important role in the muscle cell proliferation and differentiation.^17^ In chicken, IGF2 is significantly associated with broilers growth traits and carcass traits, such as birth weight, breast muscle weight, abdominal fat weight and glandular stomach weight.^18, 19, 20^

Zinc finger E-box binding homeobox 1 (ZEB1) is a crucial nuclear transcription factor, it can directly bind to the E2-box [5-CATTC(G)] sequence in different gene promoter region, repressing the activity of gene transcription.^21^ In mammal, ZEB1 has three inhibiting zones.^22^ Zone-I mainly regulates T-lymphocyte differentiation. No inhibitory activity was found in zone-II. Zone-III is associated with muscle differentiation. In chicken, ZEB1 is also called DeltaEF1 (delta-crystallin/E2-box factor 1), which is highly homologous with mice and can also recognize the E2-box sequence (CATTCG).^23^ In the process of chicken embryo development, ZEB1 was expressed in somite, notochord, urogenital system, nervous system and other parts of the chicken embryo, suggesting that it may involve in cell development process.^24^ In addition, ZEB1 can inhibit the activity of viral gene transcription by targeting the E2-box sequence exists in the promoter region of chicken infectious anemia virus, result in the decrease of the virus transcription activity.^25^

MYOD and MYOG are critical transcription factors in skeletal muscle differentiation and can regulate the transcription of most myogenesis-related genes.^26, 27, 28^ During myoblast differentiation, both of these two factors are upregulated their expression.^29, 30, 31^ MYOD and MYOG regulates their target genes transcription by binding to E-box promoter elements containing the site CANNTG.^28^ Recently, many myogenic miRNAs were found to be regulated by MYOD and MYOG,^32^ demonstrating an indirect role of these transcription factors in regulating muscle differentiation.

In this study, we investigated the function and regulation of miR-223 in avian skeletal muscle development. We found that miR-223 is regulated by MYOD transcription factor, which can bind to the promoter region of the *gga-miR-223* gene. *IGF2* and *ZEB1* are two target genes of miR-223, and the upregulation of miR-223 can inhibit myoblast proliferation and promote myoblast differentiation by its repression on these two target genes. In addition, miR-223 represses *IGF2* expression only in proliferating myoblast, and it has a more significant inhibiting effect on *ZEB1* in differentiating myoblast than in proliferating myoblast. These results identified a role for miR-223 in avian myoblast proliferation and differentiation through dynamic regulates its target genes expression, and found another regulatory pathway of MYOD to shape myoblast differentiation.

## Results

### miR-223 expression is related to skeletal muscle development

**Figure 1:**
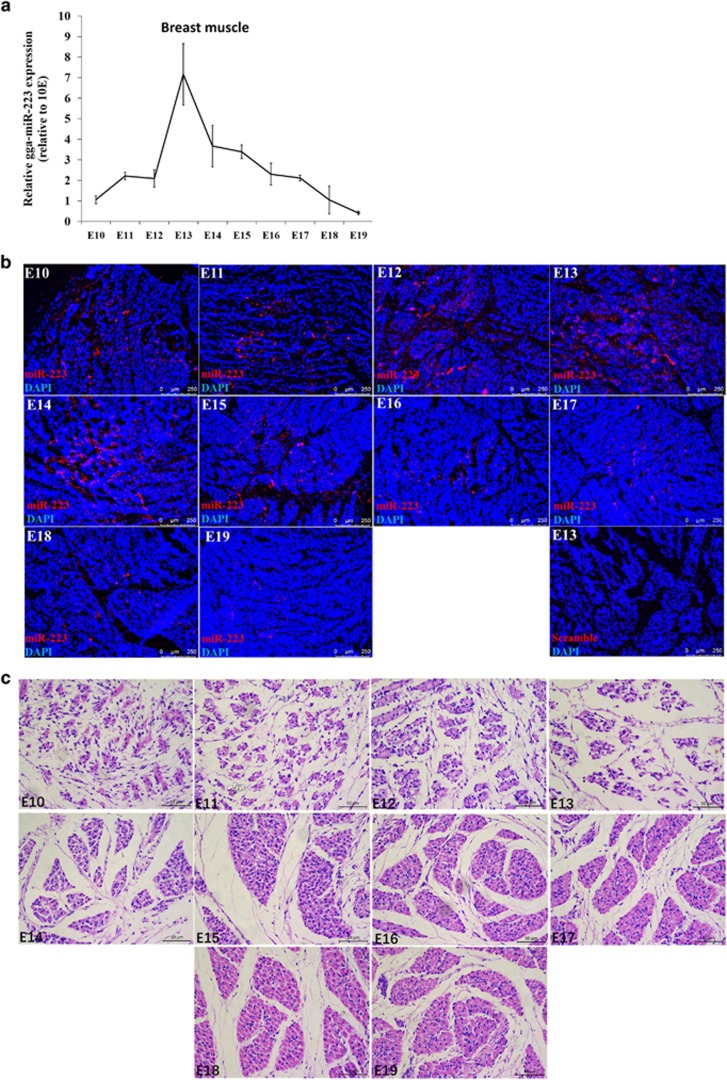
miR-223 expression is related to skeletal muscle development. (**a**) Relative expression of miR-223 in Xinghua chicken breast muscle from embryo day 10 (E10) to E19. (**b**) Fluorescence *in situ* hybridization of miR-223 in Xinghua chicken breast muscle from E10 to E19. (**c**) H-E staining of breast muscle fiber cross section from E10 to E19 in Xinghua chicken

In our previous miRNA sequencing data, we found that miR-223 exhibited differentially expression between the skeletal muscles of chickens with different growth rate.^8^ To further understand the relationship between miR-223 and skeletal muscle development, we detected its expression in breast muscle during chicken embryonic development. miR-223 was gradually upregulated its expression from embryo day 10 (E10) to E13. However, its expression was continually downregulated after E13 (Figure 1a). In addition, *in situ* hybridization results also showed that the miR-223 expression was upregulated from E10 to E13 and downregulated from E14 to E19 (Figure 1b). Haematoxylin–eosin staining of the breast muscle showed that the muscle fibers are blurry, irregular and may in the stage of cell differentiation and fusion between E10 and E13 (Figure 1c). After E13, the cross sections of muscle fibers were relatively regular and becoming more clearly defined (Figure 1c). Together with the expression data of miR-223, these results suggesting that miR-223 upregulated its expression during the active stage of muscle cell differentiation and fusion, whereas it downregulated its expression during muscle fiber maturation.

### miR-223 inhibits myoblast proliferation

**Figure 2:**
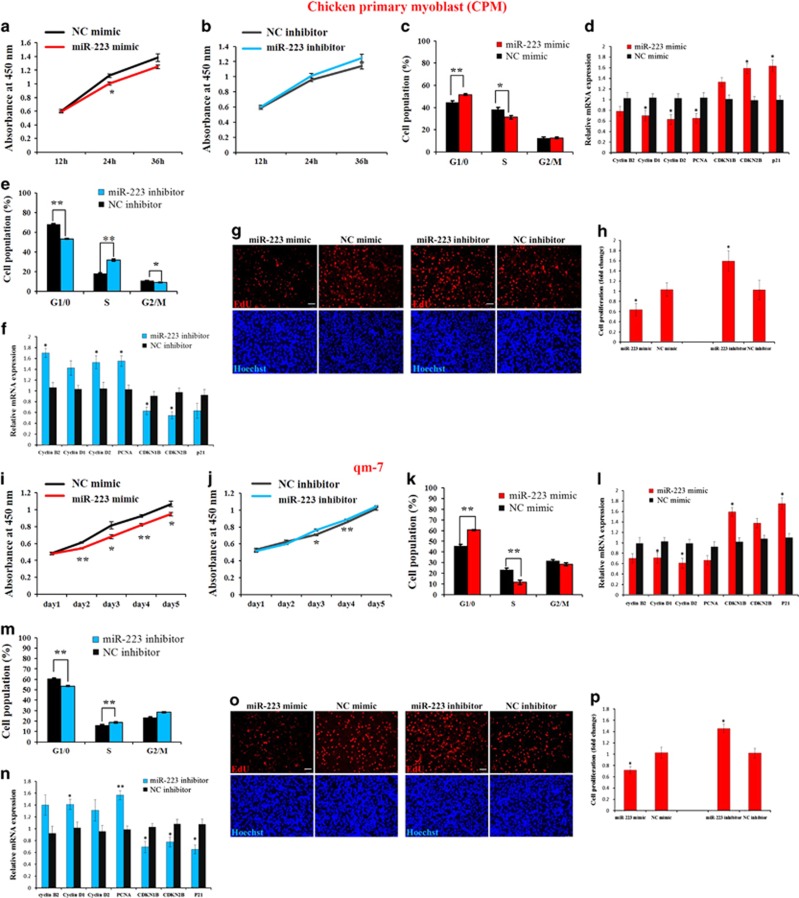
miR-223 inhibits myoblast proliferation. (**a**) CCK-8 assay indicates miR-223 overexpression inhibited chicken primary myoblast (CPM) proliferation. (**b**) CCK-8 assay indicates miR-223 inhibition promoted CPM proliferation. (**c**) CPM was overexpressed with miR-223 and the negative control (NC) mimic, and the cell cycle phase was then analyzed. (**d**) Relative mRNA expression of the cell cycle-related genes after transfection of miR-223 and NC mimic. (**e**) CPM was transfected with miR-223 inhibitor and the NC inhibitor, and the cell cycle phase was then analyzed. (**f**) Relative mRNA expression of the cell cycle-related genes after transfection of miR-223 inhibitor and NC inhibitor. (**g**) EdU staining after transfection of miR-223 mimic and miR-223 inhibitor. Bar, 50 *μ*m. (**h**) The proliferation rate of myoblasts transfected with miR-223 mimic and miR-223 inhibitor. (**i**) CCK-8 assay showed that miR-223 overexpression inhibited qm-7 proliferation. (**j**) CCK-8 assay showed that miR-223 inhibition promoted qm-7 proliferation. (**k**) qm-7 was overexpressed with miR-223 and the NC mimic, and the cell cycle phase was then analyzed. (**l**) Relative mRNA expression of the cell cycle-related genes after transfection of miR-223 and NC mimic. (**m**) qm-7 was transfected with miR-223 inhibitor and the NC inhibitor, and the cell cycle phase was then analyzed. (**n**) Relative mRNA expression of the cell cycle-related genes after transfection of miR-223 inhibitor and NC inhibitor. (**o**) EdU staining after transfection of miR-223 mimic and miR-223 inhibitor. Bar, 50 *μ*m. (**p**) The proliferation rate of qm-7 cells transfected with miR-223 mimic and miR-223 inhibitor. Results are shown as the mean±S.E.M. of three independent experiments. Independent sample *t*-test was used to analysis the statistical differences between groups. *P<0.05; **P<0.01

To further determine the roles of miR-223 in chicken skeletal muscle development, we transfected miR-223 mimics and inhibitors into chicken primary myoblast (CPM), respectively. CCK-8 assay indicated that miR-223 overexpression inhibited myoblast proliferation (Figure 2a), whereas miR-223 loss-of-function promoted myoblast proliferation (Figure 2b). In addition, by analyzing cell cycle of the transfected myoblasts, we found that miR-223 overexpression can decrease cell population in the S phase and increase cell population in the G1/0 phase (Figure 2c). miR-223 overexpression can also inhibit the expression of cell cycle-promoting genes and enhance cell cycle-inhibiting genes (Figure 2d). On the contrary, inhibition of miR-223 significantly increased cell population in the S phase and decreased cell population in the G1/0 phase (Figure 2e). miR-223 inhibition also enhanced the expression of cell cycle-promoting genes and repressed cell cycle-inhibiting genes (Figure 2f). EdU staining showed that the proliferation rate of miR-223-transfected cells was significantly reduced compared with that of the control cells (Figure 2g and h), whereas miR-223 loss-of-function promoted cell proliferation rate (Figure 2g and h). Furthermore, these roles of miR-223 were also existed in the qm-7 cells (Figure 2i and p), indicating that miR-223 can inhibit avian myoblast proliferation.

### The inhibitory effect of miR-223 on myoblast proliferation was achieved by its target gene *IGF2*

**Figure 3:**
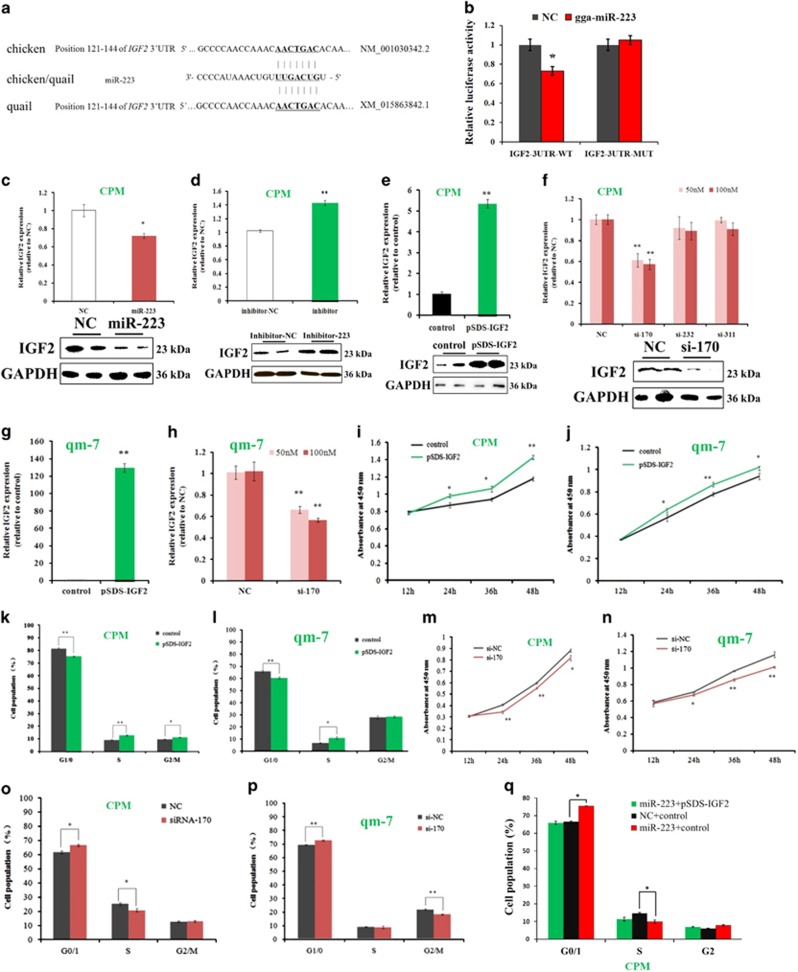
The inhibitory effect of miR-223 on myoblast proliferation was achieved by its target gene *IGF2*. (**a**) The potential binding site of miR-223 in the *IGF2* mRNA 3′ UTR. (**b**) Dual-luciferase reporter assay indicated that miR-223 can bind to the predicted binding site of the *IGF2* mRNA 3′ UTR. (**c**) miR-223 overexpression significantly inhibited *IGF2* mRNA and protein expression in CPM. (**d**) miR-223 inhibition significantly promoted *IGF2* mRNA and protein expression in CPM. (**e**) IGF2 overexpression significantly promoted *IGF2* mRNA and protein expression in CPM. (**f**) IGF2 inhibition significantly inhibited *IGF2* mRNA and protein expression in CPM. (**g**) IGF2 overexpression significantly promoted *IGF2* mRNA and protein expression level in qm-7. (**h**) IGF2 inhibition significantly inhibited *IGF2* mRNA and protein expression level in qm-7. (**i**) CCK-8 assay indicates that *IGF2* overexpression significantly promoted myoblast proliferation in CPM. (**j**) CCK-8 assay indicates that *IGF2* overexpression significantly promoted myoblast proliferation in qm-7. (**k**) CPM was overexpressed with IGF2 and the control vector, and the cell cycle phase was then analyzed. (**l**) qm-7 was overexpressed with IGF2 and the control vector, and the cell cycle phase was then analyzed. (**m**) CCK-8 assay indicates that *IGF2* inhibition significantly reduced myoblast proliferation in CPM. (**n**) CCK-8 assay indicates that *IGF2* inhibition significantly reduced myoblast proliferation in qm-7. (**o**) CPM was transfected with IGF2 siRNA and the NC siRNA, and the cell cycle phase was then analyzed. (**p**) qm-7 was transfected with IGF2 siRNA and the NC siRNA, and the cell cycle phase was then analyzed. (**q**). CPM was co-transfected with miR-223 and pSDS-IGF2, miRNA mimic NC and control vector, miR-223 and control vector, respectively, and the cell cycle phase was then analyzed. Results are shown as the mean±S.E.M. of three independent experiments. Independent sample *t-*test was used to analysis the statistical differences between groups. *P<0.05; **P<0.01

IGF2 is an important growth factor that can functioning as growth promoting hormone during cell development. Here, we found that the 3′-untranslated regions (3′-UTR) of *IGF2* mRNA has a potential binding site of miR-223 (Figure 3a). To validate whether *IGF2* is a target gene of miR-223, we constructed two dual-luciferase reporters with the wide-type and mutant 3′-UTR of *IGF2*, respectively. Results shown that miR-223 significantly repressed the luciferase activity of the wide-type reporter, whereas it has no effect on the luciferase activity of the mutant reporter (Figure 3b). In addition, miR-223 significantly inhibited the mRNA and protein expression of *IGF2* gene in CPM (Figure 3c), and the inhibition of miR-223 released *IGF2* mRNA and protein expression (Figure 3d). The above results indicated that *IGF2* is a direct target gene of miR-223.

Although *IGF2* is a well-known growth promoting-gene, its roles in chicken myoblast proliferation have not been examined. In this study, we transfected pSDS-IGF2 and three IGF2 siRNAs in poultry myoblast to overexpressing or inhibiting IGF2 expression, respectively. pSDS-IGF2 transfection significantly increased *IGF2* mRNA and protein expression, whereas the si-170, which is specifically designed for chicken *IGF2* mRNA, significantly repressed *IGF2* mRNA and protein expression (Figure 3e and h). *IGF2* overexpression promotes myoblast proliferation through CCK-8 assay (Figure 3i and j) and cell cycle analysis (Figure 3k and l), whereas *IGF2* inhibition repressed myoblast proliferation (Figure 3m and p). Furthermore, the rescue assay shown that miR-223 overexpression can inhibit the myoblast proliferation, and the transfection of *IGF2* overexpression vector could counteract the inhibitory effect of miR-223 on myoblast proliferation (Figure 3q). Together, these results argue that the inhibitory effect of miR-223 on myoblast proliferation was achieved by its target gene *IGF2*.

### miR-223 promotes myoblast differentiation

**Figure 4:**
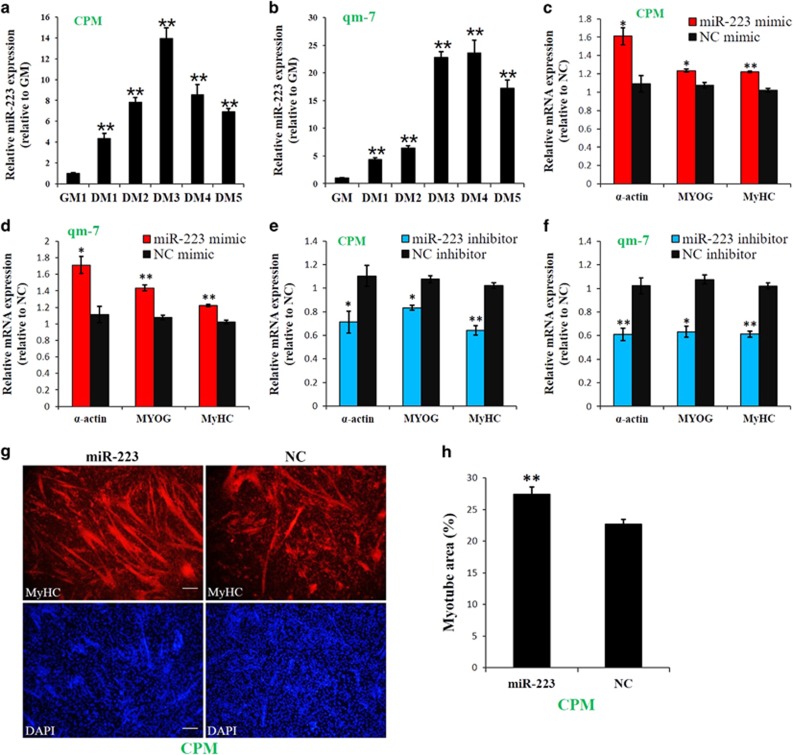
miR-223 promotes myoblast differentiation. (**a**) Relative miR-223 expression during CPM differentiation. (**b**) Relative miR-223 expression during qm-7 differentiation. (**c**) Relative expression of muscle differentiation marker genes after CPM transfected with miR-223 and NC. (**d**) Relative expression of muscle differentiation marker genes after qm-7 transfected with miR-223 and NC. (**e**) Relative expression of muscle differentiation marker genes after CPM transfected with miR-223 inhibitor and NC inhibitor. (**f**) Relative expression of muscle differentiation marker genes after qm-7 transfected with miR-223 inhibitor and NC inhibitor. (**g**) MyHC staining of CPM at 72 h after transfection of miR-223 or NC mimic. (**h**) Myotube area (%) at 72 h after transfection of miR-223 or NC mimic. Results are shown as the mean±S.E.M. of three independent experiments. Independent sample *t-*test was used to analysis the statistical differences between groups. *P<0.05; **P<0.01

miR-223 was upregulated its expression during the active stage of chicken skeletal muscle differentiation and fusion. Next, we examined the expression and function of miR-223 during myoblast differentiation and fusion. miR-223 expression was significantly upregulated from the proliferation to the differentiation of both CPM and qm-7 cells (Figure 4a and b), suggesting its involvement in these processes. Therefore, we transfected miR-223 mimic and inhibitor into the myoblasts. After transfection, the cells were induced to differentiation and fusion. Overexpression of miR-223 significantly increased the expression of muscle differentiation marker genes (Figure 4c and d), whereas the inhibition of miR-223 decreased the expression of these marker genes (Figure 4e and f). In addition, miR-223 promotes the formation of myotubes (Figure 4g), and the size of the myotube area was significantly increased after transfection of miR-223 (Figure 4h). Therefore, these results demonstrated that miR-223 can promote myoblast differentiation.

### *ZEB1* is another miR-223 target gene, functioning as an inhibitor of myoblast differentiation

**Figure 5:**
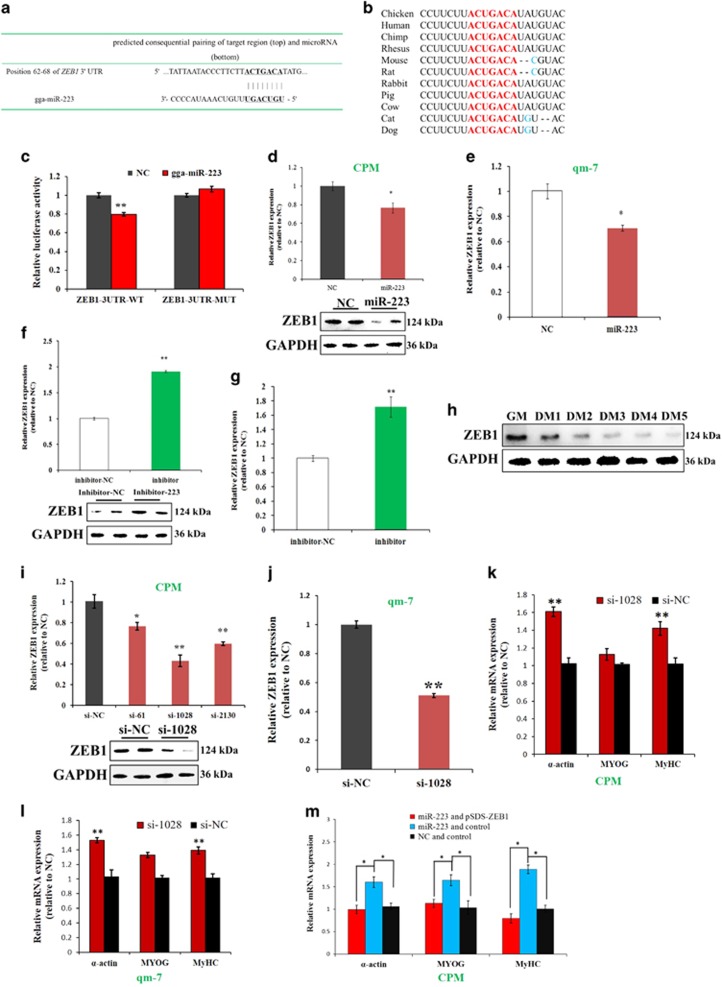
*ZEB1* is another miR-223 target gene, functioning as an inhibitor of myoblast differentiation. (**a**) The potential binding site of miR-223 in the *ZEB1* mRNA 3′ UTR. (**b**) The potential binding site (red) of miR-223 in the *ZEB1* mRNA 3′ UTR is highly conserved among vertebrates. (**c**) Dual-luciferase reporter assay indicated that miR-223 can bind to the predicted binding site of the *ZEB1* mRNA 3′ UTR. (**d**) miR-223 overexpression inhibited *ZEB1* mRNA and protein expression in CPM. (**e**) miR-223 overexpression inhibited *ZEB1* mRNA expression in qm-7. (**f**) miR-223 inhibition promoted *ZEB1* mRNA and protein expression in CPM. (**g**) miR-223 inhibition promoted *ZEB1* mRNA expression in qm-7. (**h**) ZEB1 protein expression during CPM differentiation. (**i**) ZEB1 inhibition reduced *ZEB1* mRNA and protein expression in CPM. (**j**) ZEB1 inhibition reduced *ZEB1* mRNA expression in qm-7. (**k**) Expression level of muscle differentiation marker genes after CPM transfected with *ZEB1* siRNA. (**l**) Expression level of muscle differentiation marker genes after qm-7 transfected with *ZEB1* siRNA. (**m**) CPM was co-transfected with miR-223 and pSDS-IGF2, miRNA mimic NC and control vector, miR-223 and control vector, respectively, and expression level of muscle differentiation marker genes was then analyzed. Results are shown as the mean±S.E.M. of three independent experiments. Independent sample *t*-test was used to analysis the statistical differences between groups. *P<0.05; **P<0.01

By using the TargetScan online software to predict the target genes of miR-223, we found that the 3′-UTR of *ZEB1* mRNA has a potential binding site of miR-223 (Figure 5a). Notably, this binding site is conserved among vertebrates (Figure 5b). The dual-luciferase report assay suggested that miR-223 directly binds to the predicted target site of ZEB1-3′-UTR (Figure 5c). miR-223 overexpression inhibited the mRNA and protein expression level of *ZEB1* (Figure 5d and e), and the inhibition of miR-223 promoted the mRNA and protein expression level of *ZEB1* (Figure 5f and g). Therefore, the above results indicated that *ZEB1* is another target gene of miR-223.

During myoblast proliferation and differentiation, ZEB1 protein was gradually downregulated its expression (Figure 5h). To test the function of ZEB1 in myoblast differentiation, we synthesized siRNAs specifically target for chicken *ZEB1* mRNA. Transfection of these siRNAs significantly inhibited the mRNA and protein expression level of *ZEB1* in CPM and qm-7 (Figure 5i and j). In addition, ZEB1 inhibition significantly increased the expression of muscle differentiation marker genes in both of these two cells (Figure 5k and l), suggesting ZEB1 is a repressor of myoblast differentiation. Rescue assay shown that ZEB1 overexpression is able to counteract the promotion effect of miR-223 on myoblast differentiation (Figure 5m). Therefore, *ZEB1* is another miR-223 target gene that can function as an inhibitor of myoblast differentiation.

### The inhibition of miR-223 on *IGF2* and *ZEB1* is different between myoblast proliferation and differentiation

**Figure 6:**
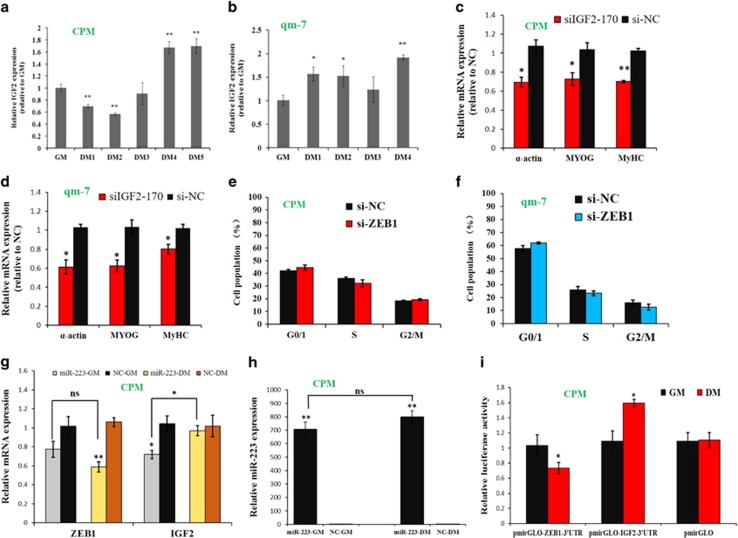
The inhibition of miR-223 on *IGF2* and *ZEB1* is different between myoblast proliferation and differentiation. (**a**) Relative *IGF2* expression level during CPM differentiation. (**b**) Relative *IGF2* expression level during qm-7 differentiation. (**c**) Expression level of muscle differentiation marker genes after CPM transfected with *IGF2* siRNA. (**d**) Expression level of the muscle differentiation marker genes after qm-7 transfected with *IGF2* siRNA. (**e**) CPM was transfected with *ZEB1* siRNA and the NC siRNA, and the cell cycle phase was then analyzed. (**f**) qm-7 was transfected with *ZEB1* siRNA and the NC siRNA, and the cell cycle phase was then analyzed. (**g**) The proliferating (GM) and differentiating (DM) CPM were transfected with miR-223 mimic, respectively, and the relative *ZEB1* and *IGF2* mRNA expression levels were then analyzed. (**h**) Relative miR-223 expression in proliferating and differentiating CPM after transfected with miR-223 and NC mimic. (**i**) The proliferating and differentiating CPM were transfected with pmirGLO-ZEB1-3′UTR, pmirGLO-IGF2-3′UTR or pmirGLO, and the relative luciferase activity was then analyzed. Results are shown as the mean±S.E.M. of three independent experiments. Independent sample *t*-test was used to analysis the statistical differences between groups. ns, no significant. *P<0.05; **P<0.01

The above results shown that both *IGF2* and *ZEB1* are target genes of miR-223. However, *IGF2* is able to promote myoblast differentiation in mammal myoblast.^33^ Its expression was increased during CPM and qm-7 differentiation (Figure 6a and b). This expression trend is similar to miR-223, which can directly inhibit *IGF2* expression. In addition, specifically inhibition of *IGF2* by siRNA significantly downregulated muscle differentiation marker genes (Figure 6c and d), indicating its promoting effect in myoblast differentiation. These results suggested that miR-223 cannot restrict IGF2 expression and function during myoblast differentiation.

On the other hand, *ZEB1*, another miR-223 target gene that can inhibit myoblast differentiation, is unable to regulate cell cycle in proliferating myoblast (Figure 6e and f). Therefore, we were interest in how the miR-223 regulates the *ZEB1* and *IGF2* expression in the proliferating and differentiating myoblast, respectively. For *ZEB1*, miR-223 inhibits its expression in both proliferation and differentiation stages of myoblast (Figure 6g), and the inhibitory effect of miR-223 on *ZEB1* has no significant difference between these two stages. For *IGF2*, miR-223 inhibits its expression only in the proliferation stage of myoblast (Figure 6g), and the miR-223 expression shown no significant difference between the proliferation and differentiation stages (Figure 6h). To further validate these results, we transfected dual-luciferase reporter inserted with the 3′-UTR of *ZEB1* or *IGF2* into the proliferating and differentiating myoblasts, respectively. The reporter activity inserted with ZEB1-3′-UTR was significant lower in differentiating myoblasts compared with that in proliferating myoblasts (Figure 6i). However, the reporter activity inserted with IGF2-3′-UTR was significant higher in differentiating myoblasts compared with that in proliferating myoblasts (Figure 6i). Together, these results suggested that the inhibition of miR-223 on *IGF2* and *ZEB1* is different between myoblast proliferation and differentiation.

### MYOD regulates *miR-223* transcription by binding to the E-box 1 region

**Figure 7:**
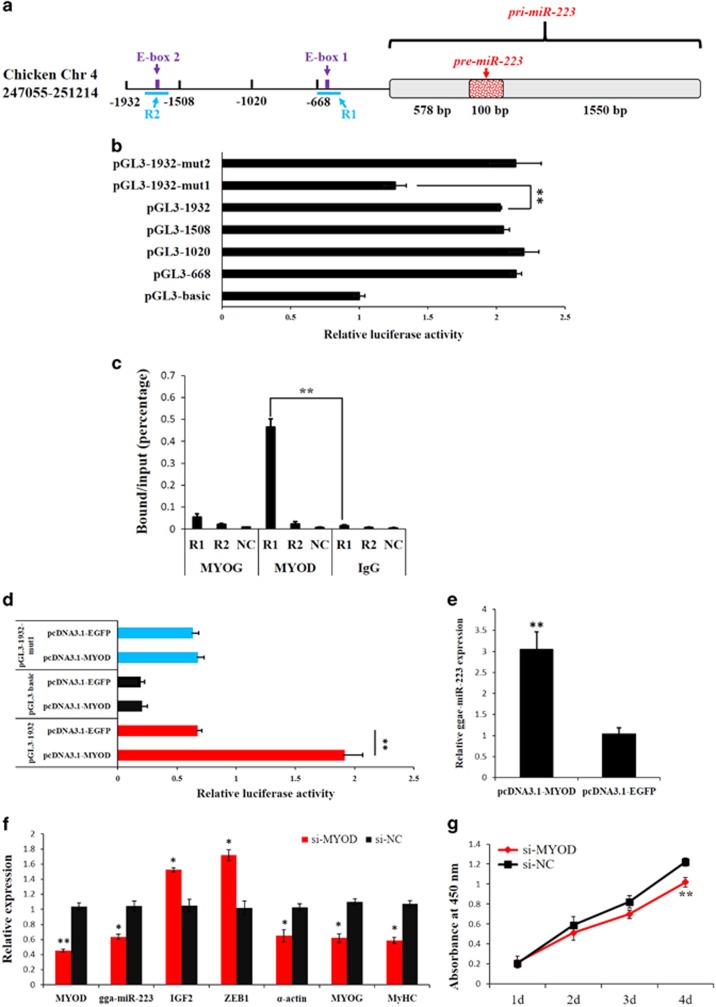
MYOD regulates miR-223 transcription by binding to the E-box 1 region. (**a**) The location of the two E-boxes in the 1932-kb upstream region of the *gga-miR-223* gene TSS. (**b**) Identification of the core region in the *gga-miR-223* gene promoter by luciferase reporter assays. (**c**) ChIP-qPCR analysis using anti-MYOG, anti-MYOD or chicken IgG antibodies, and the values showed that MYOD could bind to the R1 region of the chicken *gga-miR-223* gene in myoblasts. A region from the *GAPDH* gene was amplified as a NC to verify the specificity of the enrichment. (**d**) MYOD overexpression promoted the relative luciferase activity of the pGL3-1932 reporter in DF-1 cell. (**e**) MYOD overexpression upregulated miR-223 expression in DF-1 cell. (**f)** Relative expressions of miR-223 and its downstream genes after transfection of si-MYOD in chicken primary myoblast. (**g**) CCK-8 assay indicated that *MYOG* loss-of-function significantly reduced myoblast proliferation in chicken primary myoblast. Results are shown as the mean±S.E.M. of three independent experiments. Independent sample *t*-test was used to analysis the statistical differences between groups. *P<0.05; **P<0.01

To further understand the structure of the *gga-miR-223* gene, we isolated its full-length *pri-miR-223* by using 5′ and 3′ rapid amplification of cDNA ends (RACE). The obtained *gga-miR-223* gene was 2228 bp in length (Figure 7a). Next, we analyzed the upstream region of the *gga-miR-223* gene to find the core promotor region. Four fragments, including 668-bp, 1020-bp, 1508-bp and 1932-bp upstream regions of the *gga-miR-223* transcription start site (TSS) were amplified and cloned into the pGL3-basic vector. Forty-eight hours after transfected these reporter vectors into the myoblasts, the relative luciferase activity was measured to analyze the promoter activity of these four reporters. Results shown that the four reporters have similar relative luciferase activity (Figure 7b), suggesting that the shortest one or no reporter have the core promoter region. In addition, we found that there are two E-boxes located in the 1932-bp upstream regions of the *gga-miR-223* TSS (Figure 7a). It is well-known that the MYOG and MYOD transcription factors can bind to the E-box region and promote the transcription activity of myogenic genes.^34, 35, 36^ Considering that MYOD, MYOG and miR-223 are all upregulated their expression during myoblast differentiation, we next examined whether MYOD and MYOG can bind to these two E-box regions and promote *gga-miR-223* transcription. By mutated these two regions, respectively, we found that the relative luciferase activity was significantly reduced when we mutated E-box 1, whereas there is no change of the luciferase activity when we mutated E-box 2 (Figure 7b). This result suggesting that the E-box 1 is important for the transcription activity of the promoter region. Next, chromatin immunoprecipitation assay (ChIP) results indicated that the MYOD can bind to the E-box 1 region (Figure 7c). To avoid the effect of endogenous MYOD, we used DF-1 cell to test the regulation of MYOD on the *gga-miR-223* gene promoter and miR-223 transcription. MYOD overexpression promoted the relative luciferase activity of the pGL3-1932 reporter, but this overexpression had no effect on the luciferase activity of the mutated pGL3-1932 reporter (Figure 7d). In addition, MYOD overexpression promoted miR-223 expression (Figure 7e). For primary myoblast, MYOD loss-of-function not only inhibited the expression of miR-223 and muscle differentiation marker genes, but also promoted the expression of miR-223 target genes *IGF2* and *ZEB1* (Figure 7f). Furthermore, myoblast proliferation was also being inhibited when the cell was transfected with si-MYOD (Figure 7g). Together, these results demonstrated that the MYOD regulates *gga-miR-223* transcription by binding to the E-box 1 region.

## Discussion

**Figure 8:**
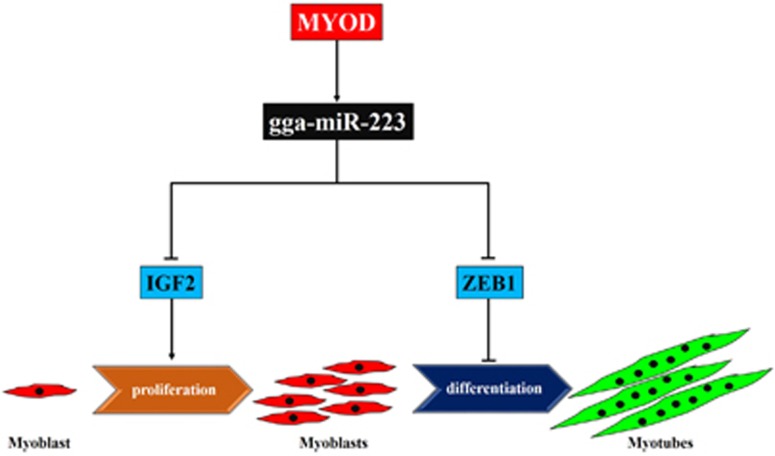
Model of the miR-223-mediated regulatory pathway for myoblast proliferation and differentiation

In this study, we identified miR-223 as another miRNA that has a role in avian skeletal muscle development. miR-223 regulates myoblast proliferation and differentiation by balancing the expression of its target genes *IGF2* and *ZEB1*. In addition, MYOD promotes miR-223 transcription during myoblast differentiation by binding to the *gga-miR-223* gene promoter region (Figure 8). These findings not only revealed a new miRNA-mediated pathway functions on skeletal muscle differentiation, but also found an interest regulatory mode of miRNA in its target genes.

MYOD belongs to the myogenic basic-helix-loop-helix transcription factors, and is an important regulator during skeletal muscle differentiation. MYOD has the ability to initiate a lot of muscle-related genes transcription by recruitment of transcription factors and histone acetyltransferases.^28, 34, 35^ Many muscle development related genes have been found to be regulated by MYOD during myoblast differentiation.^28, 34^ In this study, we found that MYOD can bind to the E-box, which is located in the promoter region of *miR-223* gene, and promote miR-223 expression in chicken myoblast. In addition, the expression of miR-223 and MYOD are upregulated during chicken myoblast differentiation,^29^ demonstrating a positive association between these two regulators. Besides, some of the muscle-specific miRNAs, such as miR-1, miR-133 and miR-206, can also be regulated by MYOD during myoblast differentiation.^37, 38^ Therefore, these findings suggested that MYOD is able to regulate myoblast differentiation through activate various myogenic miRNAs expression.

ZEB1 is a transcriptional repressor. During muscle differentiation, ZEB1 is able to repressed muscle gene transcription, and the inhibition of ZEB1 can promote myoblast differentiation and fusion.^39^ Although the repression effect of ZEB1 in muscle differentiation has been well documented, however, its expression pattern during muscle differentiation still remains unknown. In this study, we have shown that ZEB1 protein was gradually downregulated during chicken myoblast differentiation, and the decrease of ZEB1 is, at least in part, owing to the inhibition of miR-223. In addition, ZEB1 can also repress myoblast differentiation in avian, but it cannot regulate myoblast proliferation, even though it has been shown to have positive role in many cancer cell proliferation.^40, 41, 42^ MYOD can occupy G/C-centered E-boxes in the promoters of many muscle-related genes and initiate the transcription of these genes during myoblast differentiation.^43^ Interestingly, ZEB1 can also bind to the G/C-centered E-boxes in myoblast, but its binding results in the repression of muscle-related gene expression.^39^ Therefore, our results suggesting that MYOD inhibits *ZEB1* expression by promoting miR-223 expression during myoblast differentiation.

IGF2 has long been established to have important roles in muscle development,^44^ and it is also a critical growth factor for chicken.^19^ However, the function of IGF2 in avian myoblast has not been verified before. Here, we reported that IGF2 can both promote avian myoblast proliferation and differentiation, and we also found that miR-223 has the ability to inhibit *IGF2* expression. Notably, miR-223 inhibits *IGF2* expression only in the proliferating myoblast. Its inhibition effect on *IGF2* expression was significantly reduced from proliferating myoblast to differentiating myoblast. In addition, the inhibitory effect of miR-223 on *ZEB1* in the proliferating myoblast was more significant than that in the differentiating myoblast. It is generally understood that a miRNA can bind to multiple target genes, and the inhibiting effect of miRNA on these target genes would be different because of their target sites with different functional properties.^45^ However, it is rare to see that a miRNA has different binding efficiency on one target gene during cell development. Previous study has shown that synaptic stimulation can relieve the repression of miRNA to its target genes.^46, 47^ A miRNA-mediated inhibition of target gene translation can also be relieved when the cells subjected to different stress conditions, and this relief is depend on the binding of HuR, which is an AU-rich element RNA-binding protein (RBP), to the 3′UTR of the target gene.^48^ Dead end 1 (Dnd1) is another RBP evolutionary conserved among vertebrates, and also has the ability to counteracts the inhibition roles of several miRNAs by binding to the 3′UTR of target genes.^49^ However, the precise mechanism of how miR-223 dynamic regulates *IGF2* and *ZEB1* during myoblast proliferation and differentiation still need to be explored. Collectively, these and our findings suggest that the binding of miRNA to its target genes is dynamic in different cellular environment, and this binding can be rapidly responded to the specific cellular needs.^48^

In summary, this work has shown that the avian myoblast proliferation and differentiation are regulated by MYOD-miR-223-IGF2/ZEB1 pathway. In the proliferating avian myoblast, miR-223, which is upregulated by MYOD, inhibits *IGF2* expression to represses myoblast proliferation. In the differentiating avian myoblast, the upregulation of *MYOD* promotes miR-223 expression, and miR-223 inhibits *ZEB1* rather than *IGF2* to facilitate myoblast differentiation and fusion.

## Materials and methods

### Animals

The hatching eggs of Xinghua chickens were bought from the Chicken Breeding Farm of South China Agricultural University, Guangdong, China. More than ten chick embryos for each group were selected for pectorals and leg muscle separation. All the tissue samples were frozen in liquid nitrogen and stored at −80 °C for the subsequent DNA and RNA extraction. With the sex-specific primers, the sex of each embryo was determined by PCR amplification.

### RNA extraction, cDNA synthesis and quantitative real-time PCR

The total RNA was extracted from muscle tissues or cells using RNAiso reagent (Takara, Otsu, Japan). The reverse transcription reaction for mRNA was performed with PrimeScript RT reagent Kit (Perfect Real-Time) (Takara) according to manufacturer’s manual. The reverse transcription reaction for miRNA was using ReverTra Ace qPCR RT Kit (Toyobo, Osaka, Japan). The specific Bulge-loop miRNA qRT-PCR Primer for miR-223 and U6 (one RT primer and a couple of qPCR primers for each gene) were designed by RiboBio (RiboBio, Guangzhou, China). With KAPA SYBR FAST qPCR Kit (KAPA Biosystems, Wobrun, MA, USA), qPCR program was carried out in Bio-rad CFX96 Real-Time Detection system (Bio-rad, Hercules, CA, USA), and the method was as described.^50^ All reactions were run in triplicate.

### Cell culture

QM-7 cell culture: QM-7 cells were cultured in M199 (Gibco, Gaithersburg, MD, USA) supplemented with 10% fetal bovine serum (Hyclone, Logan, UT, USA), 10% tryptose phosphate broth solution (Sigma Life Science, St. Louis, MO, USA) and 0.2% penicillin/streptomycin (Invitrogen, Carlsbad, CA, USA). The differentiation of QM-7 was induced by M199 supplemented with 10% tryptose phosphate broth solution (Sigma) and 0.2% penicillin/streptomycin.

CPM isolation and culture: The leg muscle of E11 chickens were used to isolate CPM. When the skin and bones removed, the leg muscles were rapidly chopped into small pieces in petri dish containing DMEM (Gibco) media supplemented with 20% fetal bovine serum (Hyclone) and 0.2% penicillin/streptomycin. The muscle suspending liquid was shaken by vortexing 1 min and filtered to obtain single cell. Repeat the vortexing and filtering steps for 4–6 times to release enough cells. The cells were then collected by centrifugation at 1000 × *g* for 5 min at room temperature. Decant medium and discard. Add DMEM supplemented with 20% FBS and 0.2% penicillin/streptomycin to resuspend cells completely. Serial plating was needed to remove fibroblasts and enrich myoblasts. The differentiation of myoblasts was induced by DMEM supplemented with 0.2% penicillin/streptomycin.

### Immunofluorescence

The immunofluorescence was performed in CPM cultured in 24-well plates. Cells were fixed in 4% paraformaldehyde for 20 mins at room temperature and then washed three times with PBS (5 min for every turn). Subsequently, cells were treated with 0.1% Triton X-100 for 20 minutes and were blocked with goat serum for 30 min. Then, the cells were incubated with anti-MyHC (DSHB, Iowa City, IA, USA) at 4 °C overnight. After washed with PBS, the cells were treated with FITC-conjugated anti-rabbit IgG antibody and incubated in dark for 1 h. The cell nuclei were stained with DAPI (Beyotime, Jiangsu, China). The total myotube area was calculated and measured as previously described.^50^

### The 5′- and 3′-RACE

The SMARTer RACE cDNA Amplification Kit (Takara) was used to perform both 5′- and 3′-RACE according to the user manual. The 5′ and 3′ RACE-Ready first-strand cDNA was synthesized using total RNA extracted from chicken skeletal muscle. Two rounds of PCR reaction were preformed. First round was carried out with Universal Primer Mix (UPM) provided by the supplier inside the kit and miR-223 specific outer primer, and second round with Nested Universal Primer (NUP) also provided by the supplier inside the kit and miR-223 specific inner primer. Both 5′- and 3′-RACE PCR products were cloned and sequenced. All the primers used in RACE PCR are summarized in Supplementary file.

### Plasmid construction

#### pmirGLO dual-luciferase reporters

The 3′UTR fragment of *IGF2* (NCBI Reference Sequence: NM_001030342.1) and *ZEB1* (NM_205131.1) containing the binding sites were amplified by PCR from chicken embryonic leg muscle cDNA and then cloned into pmirGLO vector. The mutant vectors were constructed by PCR mutagenesis. Five seed sequences were successful mutated from ACTGA to CTGAG for IGF2-3′UTR vector, and from CTGAC to ACTGT for ZEB1-3′UTR vector.

#### Gene overexpression vector

The IGF2 overexpression vector was constructed according to the user manual of Easy Ligation Kit (Sidansai, Shanghai, China). *IGF2* coding sequence (NCBI Reference Sequence: NM_001030342.1) was amplified from chicken embryonic leg muscle cDNA by PCR. The PCR product was cloned into the pSDS-20117 vector (Sidansai). The successful IGF2 overexpression vector was confirmed by DNA sequencing. The MYOD overexpression vector was constructed as previously described.^29^

#### *miR-223* promoter reporter plasmid

A 1932-bp fragment of the *miR-223* promoter was isolated by PCR using the primers listed in Supplementary file. After the PCR product was digested with KpnI and SmaI restriction sites, the insertion was ligated into the pGL3-basic vector (Promega, Madison, WI, USA) to create the expression vector pGL3-1932. After pGL3-1932 was sequenced, this construct was used as a template, and pGL3-1508, pGL3-1020 or pGL3-668 was isolated by PCR. Site-directed mutagenesis of E-box 1 and E-box 2 was carried out by PCR amplification and DpnI digestion to remove the parental DNA.

### Transfections

Transfections were performed with Lipofectamine 3000 reagent (Invitrogen) according to the manufacturer’s direction. Nucleic acids were diluted in OPTI-MEM Medium (Gibco). All experiments were carried out at least three times independently.

### RNA oligonucleotides

The miR-223 mimics, negative control (NC), miR-223 inhibitors, miRNA inhibitor NC, siRNA against chicken *IGF2*, *ZEB1* and *MYOD* were all purchased from GenePharma (GenePharma, Shanghai, China).

### Dual-luciferase reporter assay

The miRNA target verification assay was performed in QM-7 cells. Wild-type or mutant IGF2-3′ UTR dual-luciferase reporter (200 ng) and miR-223 mimic or NC duplexes (50 nM) were co-transfected into QM-7 cells using the Lipofectamine 3000 reagent (Invitrogen) in 48-well plates. For the promoter assays, qm-7 cells and DF-1 cells were co-transfected with reporter plasmid and MYOD overexpression vector or control vector, and the TK-Renilla reporter was also co-transfected to each sample as an internal control. After 48 h transfection, cells were washed by PBS twice and the activities of Firefly and Renilla luciferase were measured according to the manual of Luc-pair Duo-Luciferase Assay Kit 2.0 (GeneCopoeia, Rockville, MD, USA). All the data were acquired by averaging the results from four independent repeats.

### Cell cycle analysis

After 36 h transfection, primary myoblast or QM-7 cells cultured in 12-well plates were fixed in 75% ethanol overnight at −20 °C. With the Cell Cycle Analysis Kit (Thermo Fisher Scientific, Waltham, MA, USA), the cells were analyzed by a BD Accuri C6 flow cytometer (BD Biosciences, San Jose, CA, USA). All the data were acquired by averaging the results from three independent experiments.

### CCK-8 assay

Primary myoblast or QM-7 cells were cultured in 96-well plates. A total of 10 *μ*l of Cell counting kit-8 reagent was added into each well and incubated for 1 h. The assay was repeated at different time points of 12, 24, 36, 48 h or every 24 h after transfection. The absorbance was measured at 450 nm by a Model 680 Microplate Reader (Bio-Rad). All the data were acquired by averaging the results from six independent experiments.

### Western blot

Western blot was performed as previously described.^50^ The following antibodies were used: anti-IGF2 (Santa Cruz Biotechnology, CA, USA), anti-ZEB1 (abcam, Cambridge, MA, USA) and anti-GAPDH (Bioworld, St Louis Park, MN, USA).

### ChIP assays

ChIP assay was performed as previously described.^29^ Samples were done in triplicate. The primer sequences for ChIP-qPCR analysis are shown in Supplementary file.

### miRNA *in situ* hybridization assay

gga-miR-223 miRCURY LNA probe and a scrambled probe were synthesized (Exiqon, Copenhagen, Denmark). 10 *μ*m thick cryosections from breast muscle tissues of Xinhua chicken were fixed in 4% PFA for 15 min at room temperature, and then treated for 10 min with Proteinase K. miRNA *in* *situ* hybridization was performed using the FISH kit (Exonbio Lab, Guangzhou, China). DNA was counterstained with DAPI (1 mg/ml). Images of miRNA signals in slides were captured by a Leica DMi8 fluorescent microscope.

### EdU assay

EdU assay was performed as previously described with the following modifications.^29^ Twelve hours after transfection, primary myoblasts were exposed to 10 *μ*M 5-ethynyl-2′-deoxyuridine (EdU; RiboBio) for 24 h at 37 °C, and the QM-7 cells were exposed to 50 *μ*M EdU for 2 h at 37 °C. In addition, for primary myoblasts, 1 × Apollo reaction cocktail (RiboBio) was added to the cells and incubated for 30 min. Whereas, in QM-7 cells, 1 × Apollo reaction cocktail was added to the cells and incubated for 20 min. The EdU-stained cells were visualized under a Leica DMi8 fluorescent microscope. The proliferation rate was calculated by the number of EDU-stained cells normalized to the number of Hoechst 33342-stained cells.

### Statistical analysis

Each experiment was repeated three times, and all results are represented as the mean±S.E.M. Independent sample *t*-test was used to perform the statistical significant difference between groups.

### Ethics standards

All experimental protocols were approved by the South China Agricultural University Institutional Animal Care and Use Committee (approval ID: SCAU#0014). And the methods were carried out in accordance with the regulations and guidelines established by this committee.

## Publisher’s Note

Springer Nature remains neutral with regard to jurisdictional claims in published maps and institutional affiliations.

## References

[bib1] Bartel DP. MicroRNAs: genomics, biogenesis, mechanism, and function. Cell 2004; 116: 281–297. 14744438 10.1016/s0092-8674(04)00045-5

[bib2] Rengaraj D, Lee BR, Lee SI, Seo HW, Han JY. Expression patterns and miRNA regulation of DNA methyltransferases in chicken primordial germ cells. PLoS ONE 2011; 6: e19524. 21559294 10.1371/journal.pone.0019524 PMC3086922

[bib3] Gschwend AR, Weingartner LA, Moore RC, Ming R. The sex-specific region of sex chromosomes in animals and plants. Chromosome Res 2012; 20: 57–69. 22105696 10.1007/s10577-011-9255-y

[bib4] Kang L, Cui X, Zhang Y, Yang C, Jiang Y. Identification of miRNAs associated with sexual maturity in chicken ovary by Illumina small RNA deep sequencing. BMC Genomics 2013; 14: 352. 23705682 10.1186/1471-2164-14-352 PMC3700833

[bib5] Huang HY, Liu RR, Zhao GP, Li QH, Zheng MQ, Zhang JJ et al. Integrated analysis of microRNA and mRNA expression profiles in abdominal adipose tissues in chickens. Sci Rep 2015; 5: 16132. 26531148 10.1038/srep16132 PMC4632014

[bib6] Han B, Lian L, Li X, Zhao C, Qu L, Liu C et al. Chicken gga-miR-103-3p targets CCNE1 and TFDP2 and inhibits MDCC-MSB1 cell migration. G3 (Bethesda) 2016; 6: 1277–1285. 26935418 10.1534/g3.116.028498 PMC4856079

[bib7] Li Y, Wang X, Yu J, Shao F, Zhang Y, Lu X et al. MiR-122 targets the vanin 1 gene to regulate its expression in chickens. Poult Sci 2016; 95: 1145–1150. 26944978 10.3382/ps/pew039

[bib8] Ouyang H, He X, Li G, Xu H, Jia X, Nie Q et al. Deep sequencing analysis of miRNA expression in breast muscle of fast-growing and slow-growing broilers. Int J Mol Sci 2015; 16: 16242–16262. 26193261 10.3390/ijms160716242 PMC4519947

[bib9] Chen CZ, Li L, Lodish HF, Bartel DP. MicroRNAs modulate hematopoietic lineage differentiation. Science 2004; 303: 83–86. 14657504 10.1126/science.1091903

[bib10] Fazi F, Rosa A, Fatica A, Gelmetti V, De Marchis ML, Nervi C et al. A minicircuitry comprised of microRNA-223 and transcription factors NFI-A and C/EBPalpha regulates human granulopoiesis. Cell 2005; 123: 819–831. 16325577 10.1016/j.cell.2005.09.023

[bib11] Masaki S, Ohtsuka R, Abe Y, Muta K, Umemura T. Expression patterns of microRNAs 155 and 451 during normal human erythropoiesis. Biochem Biophys Res Commun 2007; 364: 509–514. 17964546 10.1016/j.bbrc.2007.10.077

[bib12] Yuan JY, Wang F, Yu J, Yang GH, Liu XL, Zhang JW. MicroRNA-223 reversibly regulates erythroid and megakaryocytic differentiation of K562 cells. J Cell Mol Med 2009; 13: 4551–4559. 19017354 10.1111/j.1582-4934.2008.00585.x PMC4515070

[bib13] Yang W, Lan X, Li D, Li T, Lu S. MiR-223 targeting MAFB suppresses proliferation and migration of nasopharyngeal carcinoma cells. BMC Cancer 2015; 15: 461. 26055874 10.1186/s12885-015-1464-x PMC4460644

[bib14] Rinderknecht E, Humbel RE. Primary structure of human insulin-like growth factor II. FEBS Lett 1978; 89: 283–286. 658418 10.1016/0014-5793(78)80237-3

[bib15] Vasilatos-Younken R, Scanes CG. Growth hormone and insulin-like growth factors in poultry growth: required, optimal, or ineffective? Poult Sci 1991; 70: 1764–1780. 1924095 10.3382/ps.0701764

[bib16] Darling DC, Brickell PM. Nucleotide sequence and genomic structure of the chicken insulin-like growth factor-II (IGF-II) coding region. Gen Comp Endocrinol 1996; 102: 283–287. 8804558 10.1006/gcen.1996.0071

[bib17] Gerrard DE, Okamura CS, Ranalletta MA, Grant AL. Developmental expression and location of IGF-I and IGF-II mRNA and protein in skeletal muscle. J Anim Sci 1998; 76: 1004–1011. 9581923 10.2527/1998.7641004x

[bib18] Amills M, Jimenez N, Villalba D, Tor M, Molina E, Cubilo D et al. Identification of three single nucleotide polymorphisms in the chicken insulin-like growth factor 1 and 2 genes and their associations with growth and feeding traits. Poult Sci 2003; 82: 1485–1493. 14601723 10.1093/ps/82.10.1485

[bib19] Tang S, Sun D, Ou J, Zhang Y, Xu G, Zhang Y. Evaluation of the IGFs (IGF1 and IGF2) genes as candidates for growth, body measurement, carcass, and reproduction traits in Beijing You and Silkie chickens. Anim Biotechnol 2010; 21: 104–113. 20379887 10.1080/10495390903328090

[bib20] Gholami M, Erbe M, Garke C, Preisinger R, Weigend A, Weigend S et al. Population genomic analyses based on 1 million SNPs in commercial egg layers. PLoS ONE 2014; 9: e94509. 24739889 10.1371/journal.pone.0094509 PMC3989219

[bib21] van Grunsven LA, Taelman V, Michiels C, Opdecamp K, Huylebroeck D, Bellefroid EJ. deltaEF1 and SIP1 are differentially expressed and have overlapping activities during Xenopus embryogenesis. Dev Dyn 2006; 235: 1491–1500. 16518800 10.1002/dvdy.20727

[bib22] Postigo AA, Dean DC. Differential expression and function of members of the zfh-1 family of zinc finger/homeodomain repressors. Proc Natl Acad Sci USA 2000; 97: 6391–6396. 10841546 10.1073/pnas.97.12.6391 PMC18613

[bib23] Sekido R, Murai K, Funahashi J, Kamachi Y, Fujisawa-Sehara A, Nabeshima Y et al. The delta-crystallin enhancer-binding protein delta EF1 is a repressor of E2-box-mediated gene activation. Mol Cell Biol 1994; 14: 5692–5700. 8065305 10.1128/mcb.14.9.5692-5700.1994 PMC359094

[bib24] Funahashi J, Sekido R, Murai K, Kamachi Y, Kondoh H. Delta-crystallin enhancer binding protein delta EF1 is a zinc finger-homeodomain protein implicated in postgastrulation embryogenesis. Development 1993; 119: 433–446. 7904558 10.1242/dev.119.2.433

[bib25] Miller MM, Jarosinski KW, Schat KA. Negative modulation of the chicken infectious anemia virus promoter by COUP-TF1 and an E box-like element at the transcription start site binding deltaEF1. J Gen Virol 2008; 89: 2998–3003. 19008385 10.1099/vir.0.2008/003103-0

[bib26] Berkes CA, Tapscott SJ. MyoD and the transcriptional control of myogenesis. Semin Cell Dev Biol 2005; 16: 585–595. 16099183 10.1016/j.semcdb.2005.07.006

[bib27] Braun T, Gautel M. Transcriptional mechanisms regulating skeletal muscle differentiation, growth and homeostasis. Nat Rev Mol Cell Biol 2011; 12: 349–361. 21602905 10.1038/nrm3118

[bib28] Cao Y, Kumar RM, Penn BH, Berkes CA, Kooperberg C, Boyer LA et al. Global and gene-specific analyses show distinct roles for Myod and Myog at a common set of promoters. EMBO J 2006; 25: 502–511. 16437161 10.1038/sj.emboj.7600958 PMC1383539

[bib29] Luo W, Li E, Nie Q, Zhang X. Myomaker, regulated by MYOD, MYOG and miR-140-3p, promotes chicken myoblast fusion. Int J Mol Sci 2015; 16: 26186–26201. 26540045 10.3390/ijms161125946 PMC4661805

[bib30] Davis RL, Weintraub H, Lassar AB. Expression of a single transfected cDNA converts fibroblasts to myoblasts. Cell 1987; 51: 987–1000. 3690668 10.1016/0092-8674(87)90585-x

[bib31] Hasty P, Bradley A, Morris JH, Edmondson DG, Venuti JM, Olson EN et al. Muscle deficiency and neonatal death in mice with a targeted mutation in the myogenin gene. Nature 1993; 364: 501–506. 8393145 10.1038/364501a0

[bib32] Luo W, Nie Q, Zhang X. MicroRNAs involved in skeletal muscle differentiation. J Genet Genomics 2013; 40: 107–116. 23522383 10.1016/j.jgg.2013.02.002

[bib33] Ge Y, Sun Y, Chen J. IGF-II is regulated by microRNA-125b in skeletal myogenesis. J Cell Biol 2011; 192: 69–81. 21200031 10.1083/jcb.201007165 PMC3019547

[bib34] Blum R, Vethantham V, Bowman C, Rudnicki M, Dynlacht BD. Genome-wide identification of enhancers in skeletal muscle: the role of MyoD1. Genes Dev 2012; 26: 2763–2779. 23249738 10.1101/gad.200113.112 PMC3533080

[bib35] Cao Y, Yao Z, Sarkar D, Lawrence M, Sanchez GJ, Parker MH et al. Genome-wide MyoD binding in skeletal muscle cells: a potential for broad cellular reprogramming. Dev Cell 2010; 18: 662–674. 20412780 10.1016/j.devcel.2010.02.014 PMC2910615

[bib36] Aziz A, Liu QC, Dilworth FJ. Regulating a master regulator: establishing tissue-specific gene expression in skeletal muscle. Epigenetics 2010; 5: 691–695. 20716948 10.4161/epi.5.8.13045 PMC3052885

[bib37] Rosenberg MI, Georges SA, Asawachaicharn A, Analau E, Tapscott SJ. MyoD inhibits Fstl1 and Utrn expression by inducing transcription of miR-206. J Cell Biol 2006; 175: 77–85. 17030984 10.1083/jcb.200603039 PMC2064500

[bib38] Koutsoulidou A, Mastroyiannopoulos NP, Furling D, Uney JB, Phylactou LA. Expression of miR-1, miR-133a, miR-133b and miR-206 increases during development of human skeletal muscle. BMC Dev Biol 2011; 11: 34. 21645416 10.1186/1471-213X-11-34 PMC3132729

[bib39] Siles L, Sanchez-Tillo E, Lim JW, Darling DS, Kroll KL, Postigo A. ZEB1 imposes a temporary stage-dependent inhibition of muscle gene expression and differentiation via CtBP-mediated transcriptional repression. Mol Cell Biol 2013; 33: 1368–1382. 23339872 10.1128/MCB.01259-12 PMC3624276

[bib40] Hou LK, Yu Y, Xie YG, Wang J, Mao JF, Zhang B et al. miR-340 and ZEB1 negative feedback loop regulates TGF-beta- mediated breast cancer progression. Oncotarget 2016; 7: 26016–26026. 27036021 10.18632/oncotarget.8421 PMC5041961

[bib41] Zhang Y, Liu G, Wu S, Jiang F, Xie J, Wang Y. Zinc finger E-box-binding homeobox 1: its clinical significance and functional role in human thyroid cancer. Onco Targets Ther 2016; 9: 1303–1310. 27099512 10.2147/OTT.S96723 PMC4820193

[bib42] Gu Y, Zhao Y, Zhou Y, Xie Y, Ju P, Long Y et al. Zeb1 is potwntial regulator of Six2 in the proliferation, apoptosis and migration of metanephric mesenchyme cells. Int J Mol Sci 2016; 17: pii: E1283. 10.3390/ijms17081283 PMC5000680 27509493

[bib43] Soleimani VD, Yin H, Jahani-Asl A, Ming H, Kockx CE, van Ijcken WF et al. Snail regulates MyoD binding-site occupancy to direct enhancer switching and differentiation-specific transcription in myogenesis. Mol Cell 2012; 47: 457–468. 22771117 10.1016/j.molcel.2012.05.046 PMC4580277

[bib44] Florini JR, Magri KA, Ewton DZ, James PL, Grindstaff K, Rotwein PS. "Spontaneous" differentiation of skeletal myoblasts is dependent upon autocrine secretion of insulin-like growth factor-II. J Biol Chem 1991; 266: 15917–15923. 1651927

[bib45] Brennecke J, Stark A, Russell RB, Cohen SM. Principles of microRNA-target recognition. PLoS Biol 2005; 3: e85. 15723116 10.1371/journal.pbio.0030085 PMC1043860

[bib46] Ashraf SI, McLoon AL, Sclarsic SM, Kunes S. Synaptic protein synthesis associated with memory is regulated by the RISC pathway in Drosophila. Cell 2006; 124: 191–205. 16413491 10.1016/j.cell.2005.12.017

[bib47] Schratt GM, Tuebing F, Nigh EA, Kane CG, Sabatini ME, Kiebler M et al. A brain-specific microRNA regulates dendritic spine development. Nature 2006; 439: 283–289. 16421561 10.1038/nature04367

[bib48] Bhattacharyya SN, Habermacher R, Martine U, Closs EI, Filipowicz W. Relief of microRNA-mediated translational repression in human cells subjected to stress. Cell 2006; 125: 1111–1124. 16777601 10.1016/j.cell.2006.04.031

[bib49] Kedde M, Strasser MJ, Boldajipour B, Oude VJ, Slanchev K, le Sage C et al. RNA-binding protein Dnd1 inhibits microRNA access to target mRNA. Cell 2007; 131: 1273–1286. 18155131 10.1016/j.cell.2007.11.034

[bib50] Luo W, Li G, Yi Z, Nie Q, Zhang X. E2F1-miR-20a-5p/20b-5p auto-regulatory feedback loop involved in myoblast proliferation and differentiation. Sci Rep 2016; 6: 27904. 27282946 10.1038/srep27904 PMC4901305

