# Supplementary material for: miRNA-223 upregulated by MYOD inhibits myoblast proliferation by repressing IGF2 and facilitates myoblast differentiation by inhibiting ZEB1
Source: Cell Death Dis. 2017 Oct 5;8(10):e3094–. doi: 10.1038/cddis.2017.479 (PMC5682648; doi:10.1038/cddis.2017.479)
Supplement: Supplementary Information [file cddis2017479x1.docx]

**miRNA-223 upregulated by MYOD inhibits myoblast proliferation by repressing IGF2 and facilitates myoblast differentiation by inhibiting ZEB1**

Guihuan Li^1, 2, 3, *^, Wen Luo^1, 2, *^, Bahareldin A. Abdalla^1, 2^, Hongjia Ouyang^1, 2^,

Jiao Yu^1, 2^, Fan Hu^1, 2^, Qinghua Nie^1, 2, **^, Xiquan Zhang^1, 2^

**Oligonucleotides:**

For ChIP-qPCR analysis the following primers were used:

**R1 region:**

223R1-ChIP-qPCR-F: AACTGTCAACATCCCATCT;

223R1-ChIP-qPCR-R: CCCTTCTCCCGTTATTC;

**R2 region:**

223R2-ChIP-qPCR-F: GGTCCGTGGCAGATT;

223R2-ChIP-qPCR-R: CCAAATGGCTTCCTT.

**GAPDH (NC):**

s: 5’ - ATGGCATCCAAGGAGTGA

as: 5’ - GGGAGACAGAAGGGAACAG

For cloning the following primers were used for PCR:

IGF2 CDS (reference sequence: NM_001030342.1) cloning for pSDS overexpression vector:

s: 5’- GGGGGAAGACGGAGTGATGTGTGCTGCCAGGCAGAT

as: 5’- GCCGGAAGACAATGGGCCCAGGAGATCACAAATCG

pGL3-1932 cloning: s: 5’- GGGGTACCTTGGAGTCAATGGGGGAAGC

as: 5’- TCCCCCGGGCACGGGGCAGAGATAAGAGC

pGL3-1508 cloning: s: 5’- GGGGTACCACCTCCCTGAAAGCCTGATG

as: 5’- TCCCCCGGGCACGGGGCAGAGATAAGAGC

pGL3-1020 cloning: s: 5’- GGGGTACCATGAAGATGAGGGGAGCAGC

as: 5’- TCCCCCGGGCACGGGGCAGAGATAAGAGC

pGL3-668 cloning: s: 5’- GGGGTACCGGAATAACGGGAGAAGGGGG

as: 5’- TCCCCCGGGCACGGGGCAGAGATAAGAGC

IGF2 3’UTR Fragment:

s: 5'-AGCTTTGTTTAAACTCGATTTGTGATCTCCTGG-3'

as: 5'-GGAGCTCGTTACATCCCTCAACATTTCTC-3'

ZEB1 3’UTR Fragment:

s: 5'- CGAGCTCAAAACACTCAGCCATCA -3'

as: 5'- CCGCTCGAGACAGTCCAAGGCAAGTA -3'

For mutagenesis the following primers were used for PCR:

IGF2-3’UTR-mut:

s: 5’-GCCCCAACCAAACACTGAGCACAAGCAAGGAGGGGATGGCAGCAGC

as: 5’- GCTGCTGCCATCCCCTCCTTGCTTGTGCTCAGTGTTTGGTTGGGGC

ZEB1-3’UTR-mut:

s: 5’- ATTAATACCCTTCTTAACTGTATATGTACCTTTTTAGTTTTAGAAAAC

as: 5’- GTTTTCTAAAACTAAAAAGGTACATATACAGTTAAGAAGGGTATTAAT

pGL3-1932-mut1:

s: 5’-

GTTAAAAGGAACAGGGGAGCTGGTCAACTGCACAAAGCTGGCCTTTGTGCA

as: 5’ -

TGCACAAAGGCCAGCTTTGTGCAGTTGACCAGCTCCCCTGTTCCTTTTAAC

pGL3-1932-mut2:

s: 5’-

CTGCCTTTATTCAGACTGACAGGGTTACTGTAAACCCTCTGGGGTTCGTT

as: 5’-

AACGAACCCCAGAGGGTTTACAGTAACCCTGTCAGTCTGAATAAAGGCAG

For quantitative real time RT-PCR, the following primers were used:

MYOD-qPCR (reference sequence: NM_204214.2):

s: 5’- GCTACTACACGGAATCACCAAAT

as: 5’- CTGGGCTCCACTGTCACTCA

MYOG-qPCR (reference sequence: NM_204184.1):

s: 5’- CGGAGGCTGAAGAAGGTGAA

as: 5’- CGGTCCTCTGCCTGGTCAT

MyHC-qPCR (reference sequence: ENSGALT00000001427):

s: 5’- CTCCTCACGCTTTGGTAA

as: 5’- TGATAGTCGTATGGGTTGGT

α-actin-qPCR (reference sequence: XM_015284056.1):

s: 5’- TGGTGGGTATGGGTCAG

as: 5’- ACGGTTAGCTTTGGGATT

IGF2-qPCR (reference sequence: NM_001030342.1):

s: 5’- CCTGGCCTATGCGTTGGA

as: 5’- CCTCCTGTTATTTCGTCCCAC

ZEB1-qPCR (reference sequence: NM_205131.1):

s: 5’- CCCACCAAGTGCCAATC

as: 5’- TCCTGCCTTTCGTCCTG

Cyclin B2-qPCR (reference sequence: NM_001004369.1):

s: 5’- CAGTAAAGGCTACGAAAG

as: 5’- ACATCCATAGGGACAGG

Cyclin D1-qPCR (reference sequence: NM_205381.1):

s: 5’- CAGAAGTGCGAAGAGGAAGT

as: 5’- CTGATGGAGTTGTCGGTGTA

Cyclin D2-qPCR (reference sequence: NM_204213):

s: 5’- AACTTGCTCTACGACGACC

as: 5’- TTCACAGACCTCCAACATC

PCNA-qPCR (reference sequence: NM_204170.2):

s: 5’- GTGCTGGGACCTGGGTT

as: 5’- CGTATCCGCATTGTCTTCT

CDKN1B-qPCR (reference sequence: NM_204256.2):

s: 5’- GCTGTGCTGGGCTGAA

as: 5’- GGACGAAAGGATGTGGG

CDKN2B-qPCR (reference sequence: NM_204433.1):

s: 5’- CACGGCTGCGGATGAACTAG

as: 5’- TCCGACCGAAGGAGTTGACAG

p21-qPCR (reference sequence: AF513031.1):

s: 5’- GAAGAGTTGTCCACGATAAGC

as: 5’- TTCCAGTCCTCCTCAGTCC

β-actin-qPCR (reference sequence: NM_205518.1):

s: 5’- TTGTTGACAATGGCTCCGGT

as: 5’- AACCATCACACCCTGATGTCT

For RACE-PCR, the following primers were used:

5’RACR-223 outer primer: GTGCCTCACTTGGGGTATTT.

5’RACR-223 inner primer: GTCAAACTCAGCTTGTCAAA.

UPM outer:

CTAATACGACTCACTATAGGGCAAGCAGTGGTATCAACGCAGAGT.

UPM inner: CTAATACGACTCACTATAGGGC.

NUP: AAGCAGTGGTATCAACGCAGAGT.

3’RACR-223 outer primer: AAATACCCCAAGTGAGGCAC.

3’RACR-223 inner primer: TGGCAGAGTGTCAGTTTGTC.
